# Transversus abdominis plane block versus local anesthetic infiltration for anesthetic effect in peritoneal dialysis catheter insertion: A systematic review and meta-analysis

**DOI:** 10.1097/MD.0000000000034517

**Published:** 2023-08-04

**Authors:** Qingling Qi, Zijun Zhou, Yanheng Qiao, Tong Ren, Bo Yang

**Affiliations:** a Department of Anesthesiology, First Teaching Hospital of Tianjin University of Traditional Chinese Medicine, Tianjin, China; b Department of Nephrology, First Teaching Hospital of Tianjin University of Traditional Chinese Medicine, Tianjin, China; c Department of Nephrology, National Clinical Research Center for Chinese Medicine Acupuncture and Moxibustion, Tianjin, China.

**Keywords:** local anesthetic infiltration, peritoneal dialysis catheter insertion, surgical anesthesia, transversus abdominis plane block

## Abstract

The transversus abdominis plane (TAP) block is commonly used in surgical practice for postoperative analgesia in abdominal surgery. However, numerous studies have demonstrated that TAP block is also suitable for intraoperative anesthesia of peritoneal dialysis catheter (PDC) insertion, although its efficacy and safety are still controversial. Local anesthetic infiltration (LAI) is currently the most general anesthesia strategy for PDC insertion. Consequently, we conducted this systematic review and meta-analysis to identify which anesthesia strategy is better between TAP block and LAI.

**Methods:**

A systematic and comprehensive search was conducted on 5 databases, retrieving published and registered randomized controlled trials as of March 10, 2022, comparing the anesthesia effects of TAP block and LAI. The primary outcomes are the visual analogue scale (VAS) pain score of patients at various time points in the surgery. The secondary outcomes are the VAS pain score at rest at 2 and 24 hours postoperatively, intraoperative rescue anesthesia, general anesthesia switching rate, and PD-related complications.

**Results:**

There were 9 trials with 432 patients identified. TAP block was more effective than LAI at reducing intraoperative and postoperative VAS pain scores in patients. Compared to LAI, TAP block significantly reduces the dosage of anesthetics used to rescue anesthesia during surgery, the general anesthesia switching rate, and the incidence of postoperative PD-related complications in patients.

**Conclusions:**

Our systematic review and meta-analysis proved that TAP block could be used as the primary anesthetic technique for PDC insertion, with superior anesthetic effects to LAI.

## 1. Introduction

With the increasing morbidity and mortality, chronic kidney disease (CKD) has become a global public health problem. In 2017, the number of CKD patients approached 697.5 million, and the global prevalence of CKD was 9.1%.^[1]^ As CKD progresses to end-stage renal disease (ESRD), when patients can only rely on renal replacement therapy, peritoneal dialysis is increasingly chosen. Peritoneal dialysis is progressively chosen by an increasing number of patients, particularly in developing nations, as fewer therapy expenses and policy incentives.^[2,3]^ Because ESRD patients often have coagulation abnormalities and complex cardiopulmonary diseases, and most anesthetics may cause kidney damage, neither general anesthesia nor intraspinal anesthesia is suitable as the best anesthetic strategy in peritoneal dialysis catheter (PDC) insertion. Local anesthetic infiltration (LAI), the more effective plan, has the disadvantage of insufficient analgesia, frequently requiring intraoperative recuse anesthesia or switching to general anesthesia. Moreover, LAI is equally susceptible to edema and hemorrhage at the surgical site, which may obstruct the surgical field. Consequently, searching for a safer and more effective method of anesthesia has become the primary focus of contemporary research.

Since Rafi first proposed transversus abdominis plane (TAP) block in 2001, TAP block has been used for postoperative analgesia rather than surgical anesthesia for various abdominal surgeries, which may be related to the fact that it can only provide better abdominal wall analgesia and cannot provide visceral analgesia.^[4]^ Theoretically, peritoneal dialysis catheter insertion is not associated with the possibility of visceral pain; therefore, TAP block may be the preferred anesthetic method for peritoneal dialysis catheter insertion. Lots of studies support this opinion,^[5,6]^ but while these studies demonstrate the effectiveness and safety of TAP block, they also pose a new question: Which anesthesia strategy has a better analgesic effect between TAP block and LAI?

Some randomized controlled trials (RCTs) have compared the analgesic effects of TAP block and LAI, but their results are inconsistent. They are unable to explain which method is more effective clearly. Therefore, based on existing RCTs, we conducted a systematic review and meta-analysis to investigate which anesthesia regimen is more effective in peritoneal dialysis catheter insertion.

## 2. Methods

The study followed the recommendations of the Cochrane guidelines^[7]^ for meta-analysis and also followed the Preferred Reporting Items for Systematic Reviews and Meta-Analyses (PRISMA) 2020 statement^[8]^ for refinement of details. This study was registered on the Prospero website, registration number: CRD42022326417.

### 2.1. Eligibility criteria

#### 2.1.1. Inclusion criteria.

##### 2.1.1.1. Types of studies.

RCT studies that have been published or registered with no language restrictions.

##### 2.1.1.2. Participants.

End-stage renal disease patients undergoing peritoneal dialysis catheter insertion.

##### 2.1.1.3. Interventions and comparisons.

In our study, the patients in the intervention were anesthetized with TAP block, and the patients in the comparison were anesthetized with local anesthetic infiltration.

##### 2.1.1.4. Outcomes.

###### 2.1.1.4.1. Primary outcomes.

1. Visual analogue scale (VAS) pain score at the following time points: skin incision, division of subcutaneous tissue, PDC insertion, catheter exit and incision closing.

###### 2.1.1.4.2. Secondary outcomes.

1. VAS pain score at rest at 2, 24 hours postoperatively.
2. Intraoperative rescue anesthesia.
3. Switching rate from regional anesthesia (TAP block or LAI) to general anesthesia.
4. PD-related complications.

#### 2.1.2. Exclusion criteria.

1. Abstract and full text are not available.
2. Outcome measures are not associated with our study.

### 2.2. Literature search strategy

The following databases were searched: PubMed, the Cochrane Library, Embase, China National Knowledge Infrastructure (CNKI), and WanFang Data, from inception to March 10, 2022. The search strategy was conducted using a combination of MeSH and entry terms, and the references included from all retrieved articles were also searched manually to obtain additional relevant articles. The keywords were “peritoneal dialysis,” “peritoneal dialysis catheter implantation,” “peritoneal dialysis catheter insertion,” “peritoneal dialysis catheter placement,” “peritoneal dialysis catheterization,” “nerve block,” “transversus abdominis plane block.” (The detailed search strategy can be found in Table 1.)

**Table 1:** Search strategies

Database	#	Search strategy	Results
PubMed	1	“Peritoneal Dialysis”[MeSH Terms]	27,407
	2	“peritoneal dialysis catheter implantation”[Title/Abstract] OR “PDC implantation”[Title/Abstract] OR “peritoneal dialysis catheterization”[Title/Abstract] OR “peritoneal dialysis catheter placement”[Title/Abstract] OR “PDC placement”[Title/Abstract] OR “peritoneal dialysis catheter insertion”[Title/Abstract] OR “PDC insertion”[Title/Abstract]	257
	3	#1 OR #2	27,460
	4	“Nerve Block”[MeSH Terms]	24,749
	5	“Transversus abdominis plane block”[Title/Abstract] OR “transverse abdominis plane block”[Title/Abstract] OR “tap block”[Title/Abstract]	1164
	6	#4 OR #5	25,229
	7	#3 AND #6	16
Cochrane library	1	MeSH descriptor: [Peritoneal Dialysis] explode all trees	919
	2	(peritoneal dialysis catheterization):ti,ab,kw OR (peritoneal dialysis catheter implantation):ti,ab,kw OR (peritoneal dialysis catheter placement):ti,ab,kw OR (peritoneal dialysis catheter insertion):ti,ab,kw	178
	3	(PDC implantation):ti,ab,kw OR (PDC placement):ti,ab,kw OR (PDC insertion):ti,ab,kw	22
	4	#1 OR #2 OR #3	1026
	5	MeSH descriptor: [Nerve Block] explode all trees	4508
	6	(transversus abdominis plane block):ti,ab,kw OR (transverse abdominis plane block):ti,ab,kw OR (TAP block):ti,ab,kw	1885
	7	#5 OR #6	6150
	8	#4 AND #7	11
Embase	1	“peritoneal dialysis”/exp	47,389
	2	“peritoneal dialysis catheterization”:ab,ti OR “peritoneal dialysis catheter implantation”:ab,ti OR “peritoneal dialysis catheter placement”:ab,ti OR “peritoneal dialysis catheter insertion”:ab,ti OR “pdc implantation”:ab,ti OR “pdc placement”:ab,ti OR “pdc insertion”:ab,ti	400
	3	#1 OR #2	47,507
	4	“transversus abdominis plane block”/exp	1920
	5	“transversus abdominis plane block”:ab,ti OR “transverse abdominis plane block”:ab,ti OR “tap block”:ab,ti	2011
	6	#4 OR #5	2521
	7	#3 AND #6	27
CNKI	1	(主题=腹横肌平面阻滞) OR (主题=腹横肌平面神经阻滞) OR (主题=腹横筋膜阻滞)	1182
	2	((((主题%=“腹横肌平面阻滞” or 题名%=’腹横肌平面阻滞’) OR (主题%=’腹横肌平面神经阻滞’ or 题名%=’腹横肌平面神经阻滞’)) OR (主题%=’腹横筋膜阻滞’ or 题名%=’腹横筋膜阻滞’)) AND ((((主题%=’腹膜透析置管术’ or 题名%=’腹膜透析置管术’) OR (主题%=’腹透置管术’ or 题名%=’腹透置管术’)) OR (主题%=’腹膜透析导管置入术’ or 题名%=’腹膜透析导管置入术’)) OR (主题%=’腹膜透析导管植入术’ or 题名%=’腹膜透析导管植入术’)))	23
WanFang data		主题:(腹横肌平面阻滞) or 主题:(腹横肌平面神经阻滞) or 主题:(腹横筋膜阻滞)	1656
		主题:(腹膜透析置管术) or 主题:(腹透置管术) or 主题:(腹膜透析导管置入术) or 主题:(腹膜透析导管植入术)	816
		#1 and #2	35

### 2.3. Selection of studies and data extraction

Two researchers (ZJ Z and QL Q) independently screened and reviewed the titles and abstracts of all identified studies. If the criteria stated above are met, they will be included. Then, the following data were extracted from the included studies: characteristics of the included studies (first author, publication year, anesthetic method, number of participants, age, local anesthetic injected) and data on primary and secondary outcomes. A standardized data extraction form would be used. In case of disagreement, the senior researcher (B Y) would assess the study and data and determine the final results.

In the data extraction process, when the original data included in the study were incomplete or difficult to extract, we would send 2 emails to contact the corresponding author of the article to obtain the relevant data. The study would be excluded if no response was received within 2 months. When data were not described by a mean and standard deviation or standard error of the mean and 95% confidence interval (95% CI) in an article, the above method was similarly attempted. If no response was received within 2 months, the median and interquartile range would be converted to the mean and standard deviation using the method recommended in the Cochrane Handbook.^[7]^

### 2.4. Risk of bias assessment

The risk of bias will be assessed using the Cochrane Risk of Bias Tool,^[7]^ independently assessed and analyzed by 2 researchers (YH Q and T R). Any differences would be resolved through discussion with the senior researcher (B Y).

### 2.5. Statistical analysis

The meta-analysis was performed by Review Manager software. For continuous data, mean difference (MD) or standardized mean difference was used for evaluation, and dichotomous data were evaluated by risk ratio (RR) and 95% CI. *P* < .05 was considered statistically significant. The chi-square test (α = 0.1) and the I^2^ coefficient were used to judge heterogeneity. If *P* > .1, I^2^ < 50%, low heterogeneity was considered, at which time the fixed-effects model was selected for meta-analysis; if *P* < .1, I^2^ > 50%, high heterogeneity was considered, and the random-effects model was used for meta-analysis. When high heterogeneity results exist, the source of heterogeneity needs to be further analyzed. We will perform a subgroup analysis to exclude heterogeneity caused by these factors based on clinical or methodological differences in the original articles. At the same time, sensitivity analysis was carried out by Stata14 software. If more than 10 articles were included in the study, we would further do publication bias testing to analyze the heterogeneity.

## 3. Results

### 3.1. Search results and study characteristics

**Figure 1.**
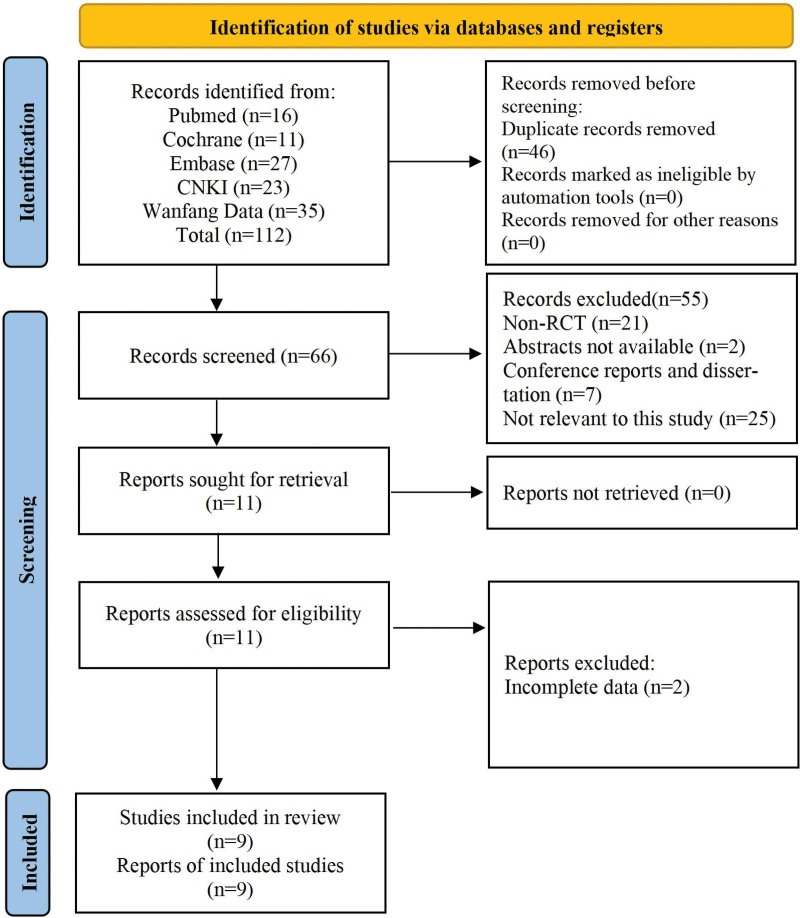
PRISMA flowchart.

In total, we retrieved 112 records that potentially met the inclusion criteria. After removing duplicates and records that did not meet the inclusion criteria, the full text was read for exclusion, 9 of which were included in our study.^[9–17]^ The specific PRISMA flowchart is shown in Figure 1.

The included studies included 432 patients (LAI: n = 198, TAP block: n = 234), and all patients were older than 18. Notably, all studies were from China. Among the included studies, one study used a bilateral TAP (Bi-TAP) block,^[15]^ one study included both unilateral TAP (Uni-TAP) block and Bi-TAP block,^[12]^ and the rest were all Uni-TAP blocks. After discussion, it was decided to subgroup the included studies into unilateral/bilateral TAP block for meta-analysis, where Li study^[12]^ was viewed as both unilateral TAP block with LAI (Li 2018-Uni) and bilateral TAP block with LAI (Li 2018-Bi). For the choice of local anesthetic drugs, one study chose lidocaine,^[13]^ one study chose a combination of lidocaine and ropivacaine,^[15]^ and the remaining studies chose ropivacaine. The detailed trial characteristics can be found in Table 2.

**Table 2:** Trial characteristics.

References	Country	Group	N (M/F)	Average age, years	Local anesthetic injected
Cai et al (2017)^[9]^	China	Uni-TAP block	10 (6/4)	52.00 ± 2.81	0.25% ropivacaine, 45 mL
		LAI	10 (7/3)	51.23 ± 2.72	0.25% ropivacaine, -mL
Dai et al (2018)^[10]^	China	Uni-TAP block	30 (18/12)	63.7 ± 11.2	0.25% ropivacaine, 25 mL
		LAI	30 (19/11)	64.5 ± 10.4	0.25% ropivacaine, -mL
Jiang et al (2021)^[11]^	China	Uni-TAP block	30 (12/18)	43.3 ± 12.7	0.25% ropivacaine, 20 mL
		LAI	30 (13/17)	41.2 ± 12.6	0.25% ropivacaine, 40 mL
Li et al (2018)^[12]^	China	Uni-TAP block	24 (14/10)	47.7 ± 11.4	0.25% ropivacaine, 40 mL
		Bi-TAP block	23 (14/9)	40.5 ± 13.3	0.25% ropivacaine, 40 mL
		LAI	22 (8/14)	41.1 ± 12.6	0.25% ropivacaine, 40 mL
Luo et al (2021)^[13]^	China	Uni-TAP block	13 (-/-)	47.50 ± 13.20	1% lidocaine, 40 mL
		LAI	14 (-/-)	50.70 ± 13.50	1% lidocaine, 35–40 mL
Ma et al (2019)^[14]^	China	Uni-TAP block	30 (17/13)	55.2 ± 4.4	0.5% ropivacaine, 25 mL
		LAI	30 (16/14)	54.9 ± 5.1	0.5% ropivacaine, 25 mL
Qiu et al (2020)^[15]^	China	Bi-TAP block	30 (15/15)	54.53 ± 15.27	1% ropivacaine, 20 mL + 2% lidocaine, 10 mL
		LAI	30 (16/14)	53.53 ± 13.99	1% ropivacaine, 20 mL + 2% lidocaine, 10 mL
Tao et al (2017)^[16]^	China	Uni-TAP block	24 (18/6)	43.75 ± 13.07	0.25% ropivacaine, 25 mL
		LAI	12 (5/7)	40.75 ± 12.31	0.25% ropivacaine, 25 mL
Wu et al (2019)^[17]^	China	Uni-TAP block	20 (7/13)	49.55 ± 15.50	0.5% ropivacaine, 25 mL
		LAI	20 (12/8)	48.35 ± 13.86	0.5% ropivacaine, 25 mL

### 3.2. Risk of bias assessments

**Figure 2.**
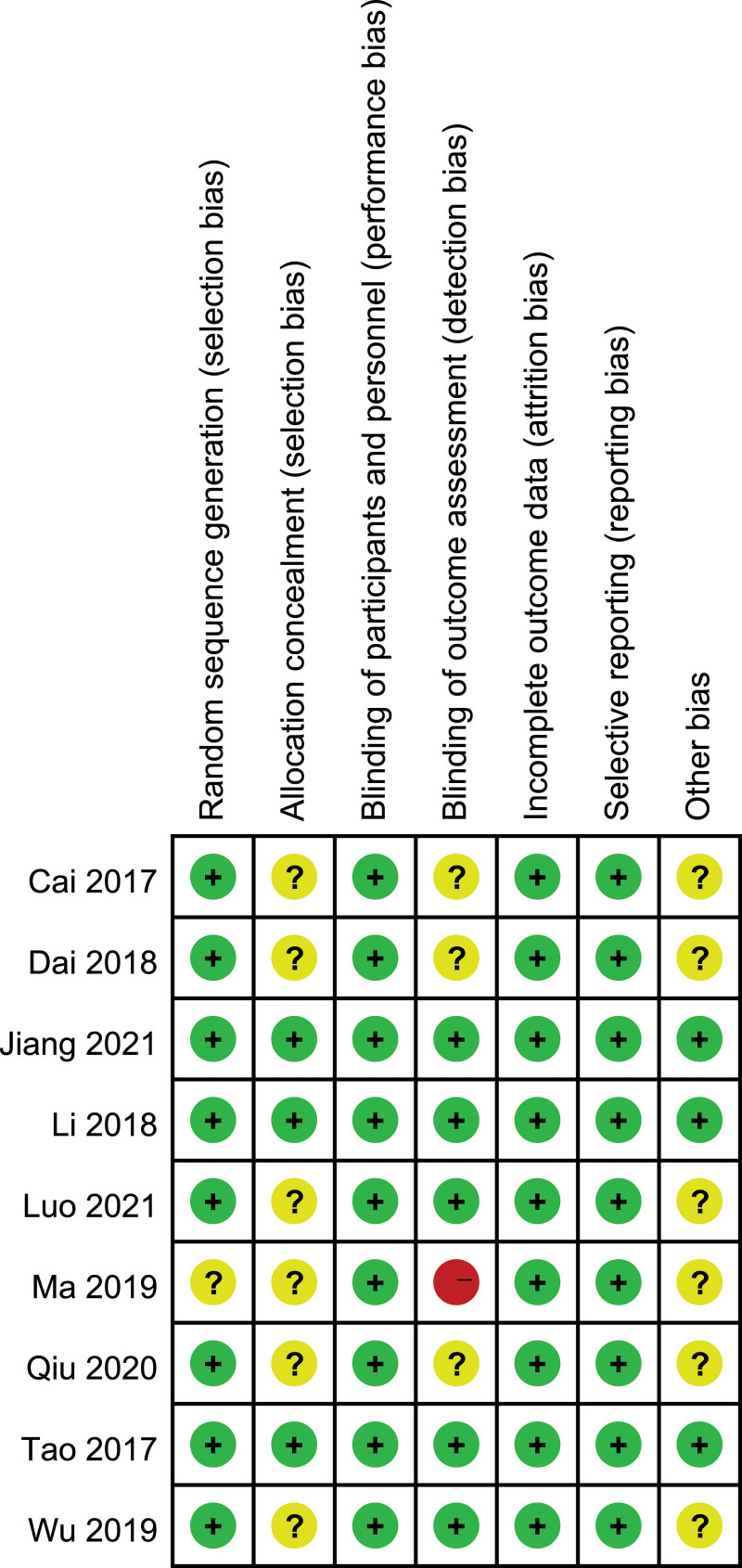
Risk of bias summary.

All RCTs included in this study were assessed for risk of bias by the Cochrane Risk of Bias Tool. Eight studies^[9–17]^ described the process of random sequence generation, of which only 3 studies^[1,12,16]^ described allocation concealment. All studies possessed a low risk of performance bias, while 3 studies^[9,10,15]^ had unclear detection bias, and one study^[14]^ had a high risk of detection bias. All studies had a low risk of attrition bias and reporting bias. In summary, the RCTs included in this meta-analysis were generally of moderate to high quality. The risk of bias for the included literature is presented in Figure 2.

### 3.3. Outcomes

#### 3.3.1. Primary outcomes.

As mentioned above, we analyzed Li study^[12]^ as 2 different studies and specific data on the outcome will be presented in Table 3.

**Table 3:** The primary outcomes

References	Country	Group	N (M/F)	VAS pain score at intraoperative and postoperative*							Rescue anesthesia†	GA	PD-related complications
				T1	T2	T3	T4	T5	T6	T7			
Cai et al (2017)^[9]^	China	Uni-TAP block	10 (6/4)	0.70 ± 0.22	/	/	/	3.78 ± 0.49	/	2.83 ± 0.65	4.2 ± 0.8*	/	/
		LAI	10 (7/3)	0.63 ± 0.71	/	/	/	6.04 ± 0.78	/	2.93 ± 0.74	18.7 ± 5.6*	/	/
Dai et al (2018)^[10]^	China	Uni-TAP block	30 (18/12)	/	/	/	4.73 ± 1.03	/	2.54 ± 0.78	/	1.76 ± 0.68	0	/
		LAI	30 (19/11)	/	/	/	6.12 ± 1.24	/	3.75 ± 1.15	/	4.20 ± 1.64	3	/
Jiang et al (2021)^[11]^	China	Uni-TAP block	30 (12/18)	3 ± 2.96	2 ± 2.41	/	5.5 ± 2.96	2 ± 2.22	1 ± 1.48	1 ± 1.48	5.3 ± 2.6	0	2
		LAI	30 (13/17)	3 ± 2.41	2 ± 2.41	/	5 ± 3.15	3 ± 3.70	1 ± 0.93	1 ± 0.74	6.5 ± 2.2	8	2
Li et al (2018)^[12]^	China	Uni-TAP block	24 (14/10)	1.2 ± 1.9	1.7 ± 1.6	2.3 ± 2.7	4.5 ± 2.8	1.0 ± 1.1	/	0.8 ± 1.0	2.5 ± 2.7	2	1
		Bi-TAP block	23 (14/9)	2.4 ± 1.7	2.5 ± 2.5	3.1 ± 2.8	4.7 ± 3.5	1.6 ± 1.8	/	1.0 ± 0.8	3.0 ± 2.8	2	1
		LAI	22 (8/14)	3.3 ± 2.7	3.2 ± 2.6	5.0 ± 3.0	3.4 ± 2.4	2.9 ± 2.3	/	1.7 ± 1.4	5.8 ± 2.6	6	3
Luo et al (2021)^[13]^	China	Uni-TAP block	13 (-/-)	3.00 ± 1.08	/	1.77 ± 0.93	1.38 ± 0.87	1.38 ± 0.87	/	/	/	/	0
		LAI	14 (-/-)	5.07 ± 0.62	/	3.71 ± 0.61	3.64 ± 0.17	3.64 ± 0.17	/	/	/	/	0
Ma et al (2019)^[14]^	China	Uni-TAP block	30 (17/13)	2.3 ± 0.7	/	2.6 ± 0.8	2.7 ± 0.9	2.7 ± 0.9	/	/	/	0	/
		LAI	30 (16/14)	2.3 ± 0.8	/	3.0 ± 0.7	3.2 ± 0.7	2.8 ± 1.0	/	/	/	0	/
Qiu et al (2020)^[15]^	China	Bi-TAP block	30 (15/15)	1.83 ± 0.79	1.40 ± 0.50	/	3.53 ± 0.86	1.37 ± 0.49	1.30 ± 0.46	/	/	0	/
		LAI	30 (16/14)	2.03 ± 0.76	1.53 ± 0.51	/	4.63 ± 1.32	1.70 ± 0.47	1.56 ± 0.50	/	/	2	/
Tao et al (2017)^[16]^	China	Uni-TAP block	24 (18/6)	2.00 ± 1.77	2.58 ± 2.19	2.39 ± 2.35	5.00 ± 2.54	2.39 ± 1.37	1.52 ± 1.20	0.70 ± 0.88	1.77 ± 2.39	1	2
		LAI	12 (5/7)	4.50 ± 2.24	4.50 ± 3.06	5.60 ± 2.46	6.38 ± 2.13	3.63 ± 1.06	2.50 ± 0.53	2.75 ± 1.49	5.21 ± 2.25	4	3
Wu et al (2019)^[17]^	China	Uni-TAP block	20 (7/13)	1.95 ± 1.39	2.45 ± 1.32	/	5.20 ± 1.54	2.80 ± 1.44	2.60 ± 1.39	/	38 ± 11.74*	/	1
		LAI	20 (12/8)	3.15 ± 1.53	3.35 ± 1.46	/	5.75 ± 1.68	3.80 ± 1.06	4.40 ± 1.57	/	54 ± 9.26*	/	5

* VAS pain score at intraoperative and postoperative: T1: skin incision, T2: division of subcutaneous tissue, T3: PDC insertion, T4: catheter exit, T5: incision closing, T6: at rest at 2 hours postoperatively, T7: at rest at 24 hours postoperatively.

† Rescue anesthesia: Those marked with * are ropivacaine (mL), and others are sufentanil (μg).

##### 3.3.1.1. VAS pain score at skin incision.

**Figure 3.**
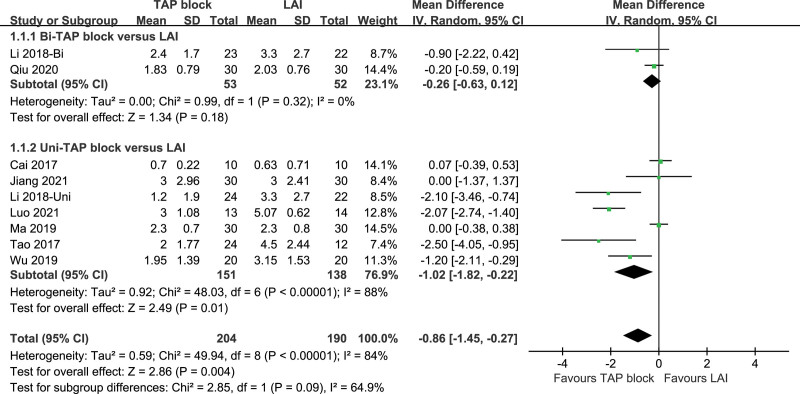
VAS pain score at skin incision. VAS = visual analogue scale.

**Figure 4.**
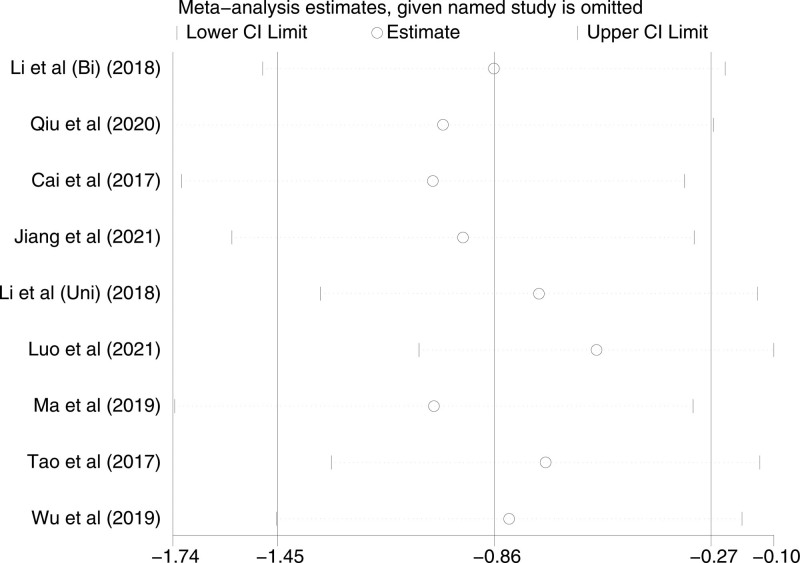
Sensitivity analysis of VAS at skin incision. VAS = visual analogue scale.

The VAS score at skin incision was recorded in 9 studies, with significant heterogeneity (*P* < 0000.1, I^2^ = 84%). A random-effects model was used. The result showed that VAS scores at skin incision of TAP block and LAI were statistically significant [*P* = .01, MD (95% CI) = −0.86 (−1.45, −0.27)]. To explain the source of heterogeneity, we performed a subgroup analysis according to the difference in unilateral/bilateral TAP block. The result showed low heterogeneity between the Bi-TAP block (*P* = .32, I2 = 0%), while the Uni-TAP block still possessed high heterogeneity (*P* < .00001, I2 = 88%). Excluding individual studies one by one for sensitivity analysis, the results did not significantly impact the overall assessment. Forest plot is shown in Figure 3, and the result of the sensitivity analysis is presented in Figure 4.

##### 3.3.1.2. VAS pain score at division of subcutaneous tissue.

**Figure 5.**
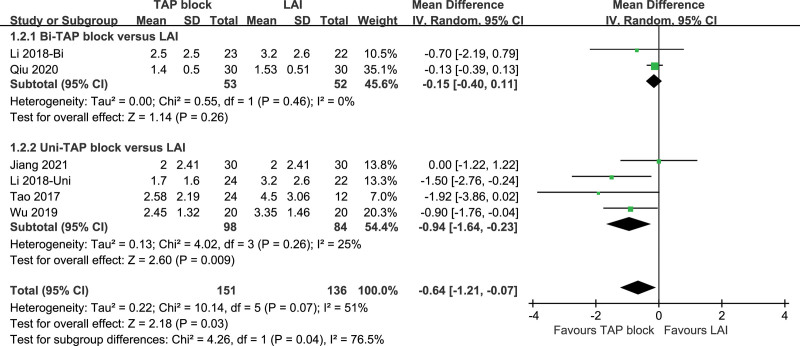
VAS pain score at division of subcutaneous tissue. VAS = visual analogue scale.

**Figure 6.**
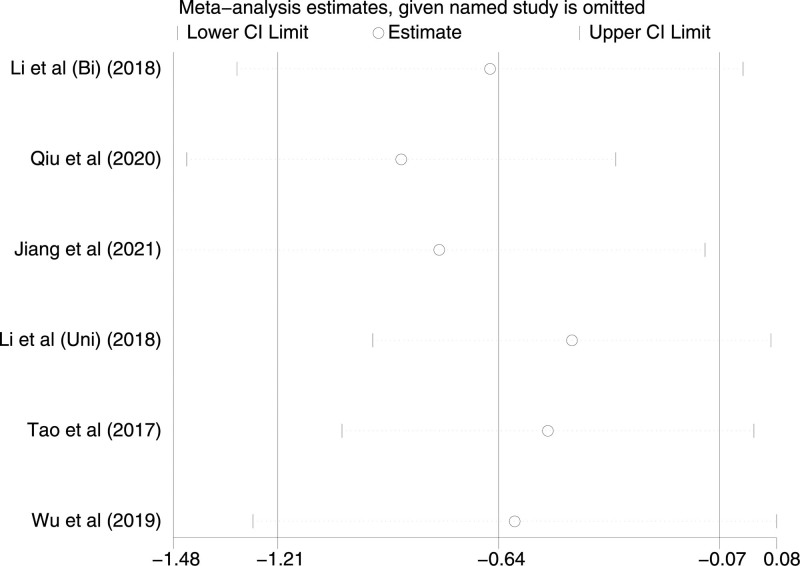
Sensitivity analysis of VAS at division of subcutaneous tissue. VAS = visual analogue scale.

Six studies recorded VAS scores at the time of the division of subcutaneous tissue. There is a moderate level of heterogeneity between them (*P* = .07, I^2^ = 51%). A random-effects model was used for the analysis, and the results of the TAP block and LAI were statistically significant [*P* = .03, MD (95% CI) = -0.64 (−1.21, −0.07)]. After performing subgroup analysis, both subgroups showed low heterogeneity (Bi-TAP block: *P* = .46, I^2^ = 0%; Uni-TAP block: *P* = .26, I^2^ = 25%). The source of heterogeneity was not found after sensitivity analysis. The Forest plot is shown in Figure 5, and the sensitivity analysis is presented in Figure 6.

##### 3.3.1.3. VAS pain score at PDC insertion.

**Figure 7.**
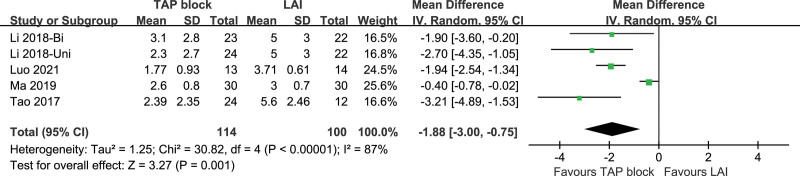
VAS pain score at PDC insertion. PDC = peritoneal dialysis catheter, VAS = visual analogue scale.

**Figure 8.**
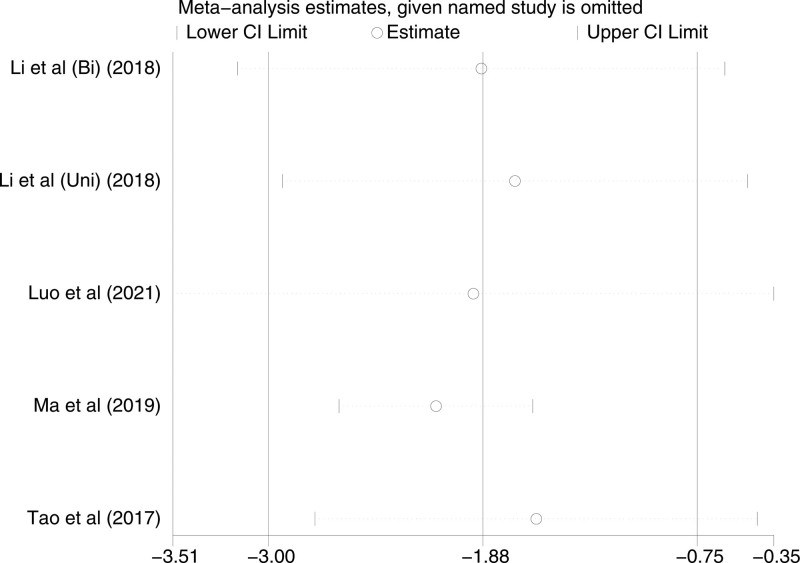
Sensitivity analysis of VAS at PDC insertion. PDC = peritoneal dialysis catheter, VAS = visual analogue scale.

Five studies included VAS scores at PDC insertion, with a high level of heterogeneity among studies (*P* < .00001, I^2^ = 82%). A random-effects model was used. There was a significant difference between TAP patients and LAI patients in terms of VAS scores at PDC insertion [*P* = .01, MD (95% CI) = −1.88 (−3.00, −0.75)]. No subgroup analysis was performed since only one of the analyzed studies was a Bi-TAP block. After performing sensitivity analyses, no studies with a significant effect on heterogeneity were identified. Forest plot is shown in Figure 7, and the sensitivity analysis is presented in Figure 8.

##### 3.3.1.4. VAS pain score at catheter exit.

**Figure 9.**
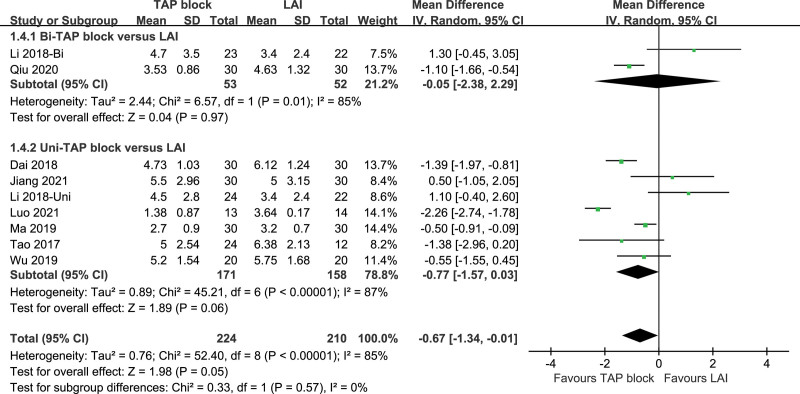
VAS pain score at catheter exit. VAS = visual analogue scale.

**Figure 10.**
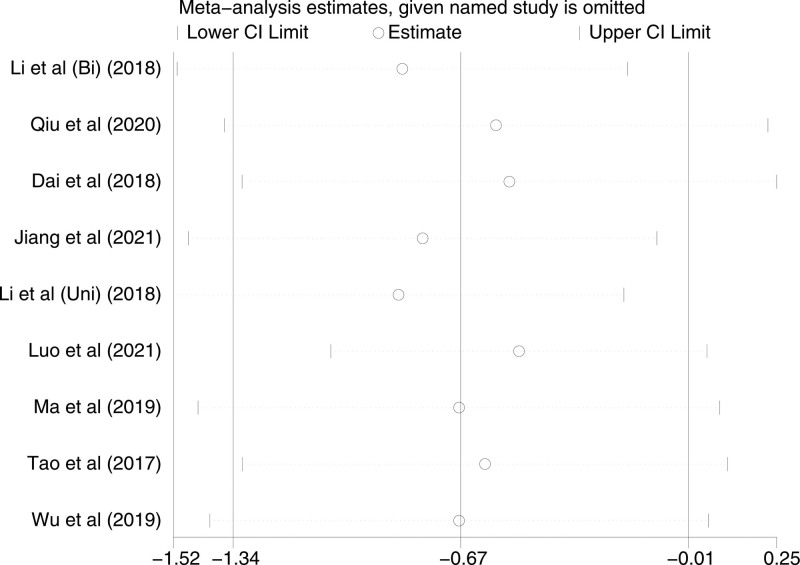
Sensitivity analysis of VAS pain score at catheter exit. VAS = visual analogue scale.

VAS scores at catheter exit were included in 9 studies, with a high level of heterogeneity in each study (*P* < .0001, I^2^ = 85%). A random-effects model was used. VAS scores at catheter exit of TAP patients and LAI patients were statistically significant [*P* = .05, MD (95%CI) = −0.67 (−1.34, −0.01)]. Subgroup analysis revealed high heterogeneity in both the Bi-TAP block and Uni-TAP block (Bi-TAP block: *P* = .01, I^2^ = 85%, Uni-TAP block: *P* < .00001, I^2^ = 87%). After performing sensitivity analyses, no studies with a significant effect on heterogeneity were identified. Forest plot is shown in Figure 9, and the sensitivity analysis is presented in Figure 10.

##### 3.3.1.5. VAS pain score at incision closing.

**Figure 11.**
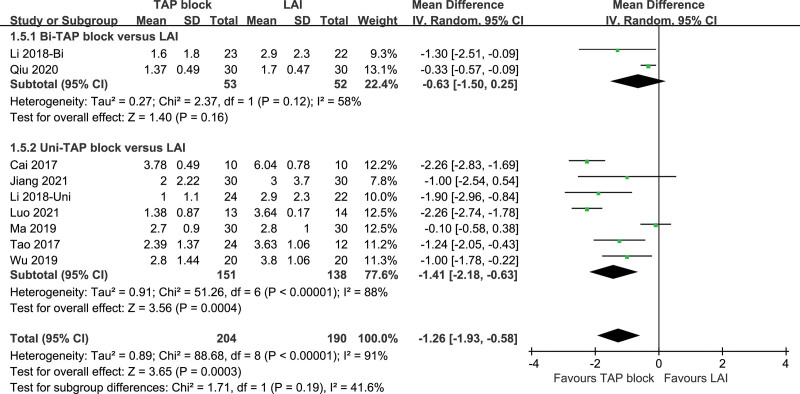
VAS pain score at incision closing. VAS = visual analogue scale.

**Figure 12.**
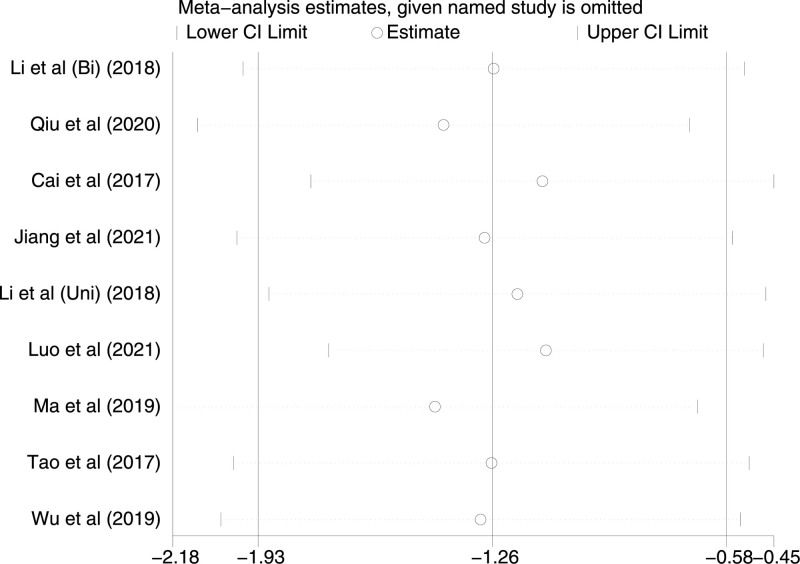
Sensitivity analysis of VAS at incision closing. VAS = visual analogue scale.

Nine studies included VAS scores at incision closing, with a high degree of heterogeneity in all studies (*P* < .0001, I^2^ = 91%). VAS scores at incision closing were statistically significant in TAP patients and LAI patients using a random-effects model [*P* = .003, MD (95% CI) = −1.26 (−1.93, −0.58)]. Subgroup analysis showed that both the Bi-TAP block and the Uni-TAP block were highly heterogeneous (Bi-TAP block: *P* = .12, I^2^ = 58%, Uni-TAP block: *P* < .00001, I^2^ = 88%). After performing sensitivity analyses, no studies with a significant effect on heterogeneity were identified. The Forest plot is shown in Figure 11, and the sensitivity analysis is presented in Figure 12.

#### 3.3.2. Secondary outcomes.

##### 3.3.2.1. VAS pain score at rest at 2 hours postoperatively.

**Figure 13.**
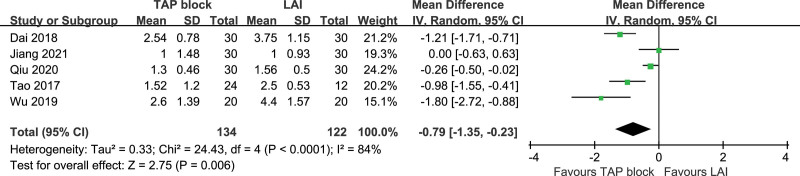
VAS pain score at rest at 2 hours postoperatively. VAS = visual analogue scale.

Five studies included VAS scores at rest at 2 hours postoperatively, and the studies were highly heterogeneous (*P* < .0001, I^2^ = 84%). A random-effects model was used. VAS scores at rest at 2 hours postoperatively were statistically significant in TAP block and LAI [*P* = .006, MD (95% CI) = −0.79 (−1.35, −0.23)]. Subgroup analysis and sensitivity analysis were not performed. Forest plot is shown in Figure 13.

##### 3.3.2.2. VAS pain score at rest at 24 hours postoperatively.

**Figure 14.**
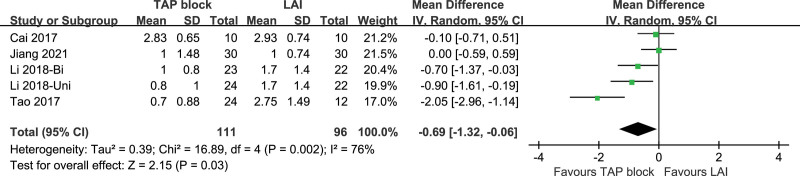
VAS pain score at rest at 24 hours postoperatively. VAS = visual analogue scale.

Five studies included VAS scores at rest at 24 hours postoperatively, and the studies were highly heterogeneous (*P* = .002, I^2^ = 76%). A random-effects model was used. VAS scores at rest at 24 hours postoperatively were statistically significant in TAP block and LAI [*P* = .003, MD (95% CI) = −0.69 (−1.32, −0.06)]. Subgroup analysis and sensitivity analysis were not performed. Forest plot is shown in Figure 14.

##### 3.3.2.3. Intraoperative rescue anesthesia.

**Figure 15.**
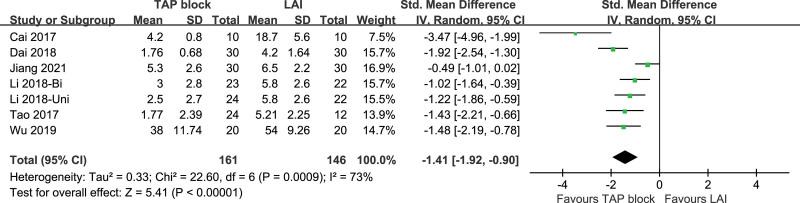
Intraoperative rescue anesthesia.

Seven studies included anesthetic drug dosages for intraoperative rescue anesthesia, 2 studies^[9,17]^ used ropivacaine, and the rest used sufentanil. These studies were highly heterogeneous (*P* = .0009, I^2^ = 73%). A random-effects model was used. The difference in the anesthetic drug dosages for intraoperative rescue anesthesia of TAP block and LAI was statistically significant [*P* < .00001, standardized mean difference (95% CI) = −1.41 (−1.92, −0.90)]. Subgroup analysis and sensitivity analysis were not performed. Forest plot is shown in Figure 15.

##### 3.3.2.4. Switching into GA.

**Figure 16.**
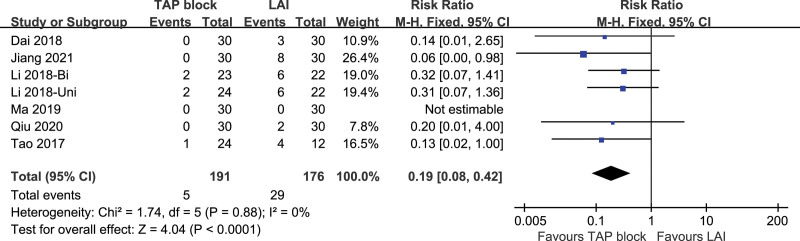
Switching into GA.

Seven studies included the switching rate from regional anesthesia (TAP block or LAI) to general anesthesia, and the studies had low heterogeneity (*P* = .88, I^2^ = 0%). A fixed-effect model was used. The difference in the switching rate from regional anesthesia to general anesthesia intraoperatively between TAP block and LAI was statistically significant [*P* < .00001, RR (95% CI) = 0.19 (0.08,0.42)]. Forest plot is shown in Figure 16.

##### 3.3.2.5. PD-related complications.

**Figure 17.**
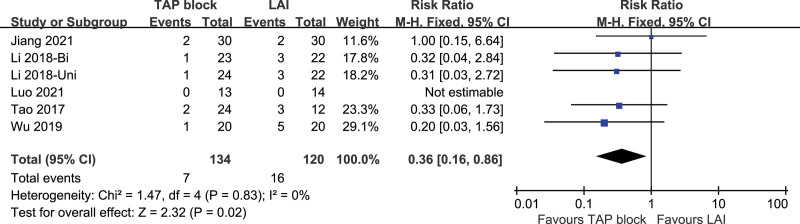
PD-related complications.

Six studies included the number of patients with postoperative PD-related complications, and the studies had low heterogeneity (*P* = .83, I2 = 0%). A fixed-effects model was used. The difference in the number of patients with postoperative PD-related complications between TAP block and LAI was statistically significant [*P* = .02, RR (95% CI) = 0.36 (0.16, 0.86)]. Forest plot is shown in Figure 17.

### 3.4. Publication bias

As no more than 10 articles were included, no publication bias test was conducted.

## 4. Discussion

Our meta-analysis is the first study to compare TAP block as the primary anesthesia technique with LAI. Of course, this is related to the particularity of peritoneal dialysis catheter insertion, which does not involve significant visceral pain during the operation. Almost all current studies focus on TAP block for postoperative pain management after abdominal surgery. Similarly, almost all meta-analyses of TAP block and LAI are exploring which is better for postoperative analgesia between the two.^[4,18,19]^

Our systematic review and meta-analysis compared the anesthesia effects of TAP block and LAI in peritoneal dialysis catheter insertion. Based on 10 studies (Li study is considered 2) RCTs with 432 patients, we demonstrated that TAP blocks had better anesthesia effects than LAI. More specifically, our results showed that TAP block could reduce patient VAS pain scores at intraoperative and postoperative time points compared to LAI. Notably, compared with LAI, the MD of VAS pain scores at skin incision and at rest at 2 hours postoperatively was close to 1 for TAP block, and the MD of VAS pain scores at PDC and the closing incision were more significant than 1. This difference indicated that the patient’s pain was significantly improved in clinical practice.^[20]^ At the same time, we also found that TAP block compared with LAI, the dosage of drugs to rescue anesthesia during surgery and general anesthesia switching rate were significantly reduced, and the incidence of postoperative PD-related complications in patients was also significantly reduced.

ESRD patients often have complex other systemic diseases, such as coagulation abnormalities, hypertension, and heart failure. The presence of these diseases makes ESRD patients more cautious when choosing an anesthesia regimen. General and intraspinal anesthesia are inappropriate as preferred regimens.^[21]^ As a commonly used method of anesthesia in clinical practice, LAI often has edema and bleeding at the surgical site during surgery, interfering with the phenomenon of the surgical field, and TAP block does not face this problem.^[22]^ TAP block targets the T7-L1 thoracolumbar nerves and is less at risk of adverse events than general anesthesia and spinal anesthesia. Especially in high-risk patients, TAP block may be the safest and most effective anesthesia technique.^[6,21]^ In our results, the effectiveness of TAP block as the primary anesthesia technique is unquestionable. Regarding VAS pain score and other aspects, TAP block is significantly superior to LAI.

In our results most of the results were significantly heterogeneous. In order to analyze the sources of heterogeneity, we conducted subgroup and sensitivity analyses for studies with significant heterogeneity in the primary outcomes. However, neither the subgroup nor the sensitivity analysis results indicate the source of heterogeneity. We note that high heterogeneity of results is widespread in other similar studies,^[18,19]^ and we analyze the sources of our analysis of this heterogeneity may be the following ways. First, high heterogeneity may be associated with the subjectivity of VAS pain scores because many factors can influence VAS pain scores in the clinic. Second, the doctors who perform TAP block are different. Their anesthesia techniques and experiences are not exactly the same, and the dosage of anesthetic drugs is also different, which may also be the cause of high heterogeneity. Finally, data processing can also be a source of heterogeneity, Jiang study^[11]^ used quartiles to record this data. Although we applied the conversion method recommended by the Cochrane Handbook, it was still not accurate to convert this data into means and standard deviations.

Although our study proved that TAP block might be more suitable for PDC insertion in ESRD patients than LAI, some limitations remain. Although the RCTs we included were high quality, the total number of included studies and sample size were relatively small, especially since all included studies were from the same country. As mentioned above, excluding heterogeneity in our results is challenging. Although we have analyzed these heterogeneities, they may still affect our conclusions. We did not analyze the operation time and economic cost. Although these factors do not affect our conclusion, they often affect the decision-making of doctors and patients in clinical practice.

While our study proved that the TAP block might be a better anesthesia technique for PDC insertion in ESRD patients than LAI, there are still some limitations: Although the quality of the RCTs we included was high, the total number of included studies the sample size was relatively small. Especially all the included studies were from the same country. As mentioned above, there are heterogeneities in our results that are difficult to exclude, and although we have analyzed these heterogeneities, they may still affect our conclusions. On the issue of surgery time and economic cost, we did not analyze them. Although these factors will not affect our conclusions, the clinic often affects the decision-making of doctors and patients.

## 5. Conclusion

Our meta-analysis showed that TAP block could be used as the primary anesthesia technique for PDC insertion, with anesthesia effects superior to LAI. TAP blocks can significantly reduce the VAS pain score of patients at various time points in the surgery, reduce the dosage of intraoperative rescue anesthetic drugs and the switching rate of general anesthesia, and are also better than LAI in postoperative analgesia and reduction of postoperative PD-related complications. In short, TAP block are more secure and effective compared to LAI. Whether it can be promoted in the clinic needs to be further verified by large-scale, high-quality RCTs.

## Author contributions

**Writing – original draft:** Qingling Qi, Zijun Zhou, Yanheng Qiao, Tong Ren, Bo Yang.
